# Bacterial Community in the Skin Microbiome of Frogs in a Coldspot of Chytridiomycosis Infection

**DOI:** 10.1007/s00248-020-01669-5

**Published:** 2021-01-13

**Authors:** Milind C. Mutnale, Gundlapally S. Reddy, Karthikeyan Vasudevan

**Affiliations:** Laboratory for the Conservation of Endangered Species, CSIR-Centre for Cellular and Molecular Biology, Hyderabad, Telangana India

**Keywords:** Amphibia, Skin microbiome, Anti-*Bd* bacteria, 454 next-generation sequencing, Endemic frogs, Proteobacteria

## Abstract

Chytridiomycosis is a fungal disease caused by the pathogens, *Batrachochytrium dendrobatidis* (*Bd*) and *B. salamandrivorans* (*Bsal*), which has caused declines in amphibian populations worldwide. Asia is considered as a coldspot of infection, since adult frogs are less susceptible to *Bd*-induced mortality or morbidity. Using the next-generation sequencing approach, we assessed the cutaneous bacterial community composition and presence of anti-*Bd* bacteria in six frog species from India using DNA isolated from skin swabs. All the six frog species sampled were tested using nested PCR and found *Bd* negative. We found a total of 551 OTUs on frog skin, of which the bacterial phyla such as Proteobacteria (56.15% average relative abundance) was dominated followed by Actinobacteria (21.98% average relative abundance) and Firmicutes (13.7% average relative abundance). The contribution of Proteobacteria in the anti-*Bd* community was highest and represented by 175 OTUs. Overall, the anti-*Bd* bacterial community dominated (51.7% anti-*Bd* OTUs) the skin microbiome of the frogs. The study highlights the putative role of frog skin microbiome in affording resistance to *Bd* infections in coldspots of infection.

**Supplementary Information:**

The online version contains supplementary material available at 10.1007/s00248-020-01669-5.

Over a decade, amphibians’ (frogs, caecilians, and salamanders) skin microbiome has been studied extensively to understand their role in the amphibian panzootic disease, called chytridiomycosis [1–3]. The disease is caused by two aquatic fungal pathogens: *Batrachochytrium dendrobatidis* (*Bd*) infects frogs, salamanders, and caecilians [4], and *B. salamandrivorans* (*Bsal*) infects both frogs and salamanders [5, 6]. They have caused declines in over 500 amphibian species, and possible extinctions of 90 species, worldwide [7]. In adult frogs, the skin microbiome plays an important role in the host’s defense mechanism [8]. The frog’s skin microbiome produces a plethora of anti-fungal metabolites to protect the host [2, 8], and the bacterial community on the skin might confer resistance to pathogens [9]. Skin microbiomes of frogs have revealed some important anti-*Bd* bacteria, such as *Janthinobacterium lividum* (family Oxalobacteraceae) [2, 8] and *Serratia marcescens* (family: Yersiniaceae) that produce anti-fungal metabolites: “Violacein” [8] and “Prodigiosin,” respectively. Both violacein and prodigiosin compromise the integrity of the cell membrane [10, 11], and prodigiosin also inhibits RNA and protein synthesis in bacteria [12]. Consequently, these two metabolites are known to inhibit *Bd* fungal growth [2]. Since anti-*Bd* bacteria are present on frogs that show resistance to *Bd* [13], investigating such skin microbiomes for potential anti-*Bd* bacterial isolates would benefit disease mitigation strategies.

There are over 395 frog species in India, and the list is growing, as new taxa are being discovered rapidly (see, http://amphibiaweb.org/). With high species richness and endemism, the stakes are high for understanding the role of *Bd* on frog populations, and the mechanisms by which frogs might be able to resist *Bd* infection. Frog populations in the region exhibit low *Bd* prevalence and high *Bd* haplotype diversity, which points at possible historical host-pathogen co-evolution [14]. Low prevalence and low mortality could also imply that frogs show some resistance to chytridiomycosis, and this could be mediated by the skin microbiome. Investigating the role of frog’s skin microbiome in coldspots of *Bd* infection could reveal new pathways that inhibit the pathogen. Therefore, we studied the bacterial community composition in six frog species from two hotspots for amphibian species richness, namely the Western Ghats and the Andaman Islands. We explored the frog skin microbiome data to infer the role of bacterial community in affording them with resistance to *Bd* infections. (Full details on the methods are provided in the Supplementary file S1.)

We generated a total of 18,543 good quality sequences from 18 frogs belonging to six species that had no *Bd* infection (Table 1). We aligned all good quality sequences in mothur and assigned them identity using SILVA v123 database. In all, we identified 1351 unique sequences (at ≥ 97% sequence similarity). These unique sequences revealed 551 operational taxonomic unit (OTU), belonging to five major bacterial phyla. Average relative abundance (ARA) of bacterial taxa in the microbiome was calculated as percentage of the total good quality bacterial sequences obtained. Proteobacteria (56.15% ARA) was the most dominant phylum, followed by Actinobacteria (21.98% ARA), Firmicutes (13.7% ARA), Bacteroidetes (7.2% ARA), and Acidobacteria (0.49% ARA). Acidobacteria was present only in *B. beryet* (1.99% relative abundance) and in *Limnonectes doriae* (0.99% relative abundance), from the Andaman Islands. In the case of aquatic *Nyctibatrachus poocha*, Proteobacteria (6.98% relative abundance) was found to be the least abundant, and Actinobacteria (49.12% relative abundance) was the most abundant (Fig. 1b). Rarefaction revealed that *L. doriae* had the highest and *Ghatixalus asterops* had the least number of OTUs (Table 1). Cluster analysis revealed that the bacterial community did not form clusters based on the niche occupied by the frogs (Fig. 1a).

**Table 1:** Details of samples collected and the data generated on skin microbiome of frogs

S. no.	Niche	Species	Number of samples pooled	Number of sequence reads generated	Total number of OTUs (standard error±)	Anti-*Bd* OTUs (%)	Sample location	Coordinates		Prevalence*
1	Arboreal	*Blythophryne beryet*	3	1210	54.7 (3.21)	47.7	Little Andaman, India	10° 44′ 47.1″ N	92° 32′ 18.5″ E	0% (0–48)
2	Terrestrial	*Duttaphrynus melanostictus*	2	6148	46.3 (4.29)	58.9	Munnar, Kerala, India	10° 04′ 24.5″ N	77° 02′ 02.5″ E	15.8% (5–37)
			1				Little Andaman, India	10° 35′ 07.0″ N	92° 33′ 29.0″ E	
3	Arboreal	*Ghatixalus asterops*	3	8176	45.0 (4.22)	79.1	Munnar, Kerala, India	10° 05′ 37.3″ N	77° 02′ 43.2″ E	12.1% (6–22)
4	Terrestrial	*Limnonectes doriae*	3	545	86.8 (2.81)	26.5	Middle Andaman, India	11° 36′ 48.5″ N	92° 36′ 43.9″ E	8.2% (5–12)
5	Aquatic	*Nyctibatrachus poocha*	3	2043	50.5 (3.86)	19.8	Munnar, Kerala, India	10° 04′ 24.5″ N	77° 02′ 02.5″ E	16.7% (6–40)
6	Arboreal	*Raochestes chlorosomma*	3	421	57.0 (N/A)	73.7	Munnar, Kerala, India	10° 04′ 24.5″ N	77° 02′ 02.5″ E	6.3% (0.3–28)

*N/A*, not computed

*Numbers represent prevalence with 95% CI of that species population across the country. Prevalence data was obtained from a previous study by Mutnale et al. [14]

**Fig. 1:**
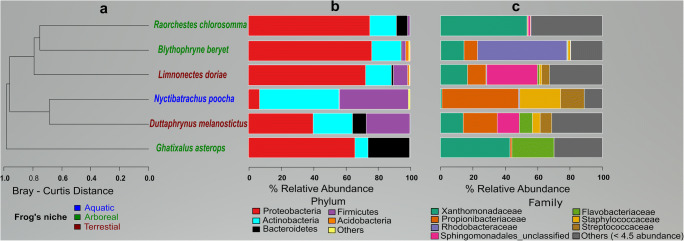
Graph represents the cluster analysis and the relative abundance of the frog skin microbiome: **a** cluster analysis of frog species is calculated using Bray-Curtis method. Font color of species name indicates niche of frog. **b** Bacterial phylum-level relative abundance in six frog species. **c** bacterial Family level relative abundance in six frog species (Plots were prepared using R (v3.6.3) software, and modified using Inkscape 0.91 (https://inkscape.org/en/))

Out of the 551 OTUs identified, 285 OTUs (51.7%) were anti-*Bd* bacterial OTUs, and they constituted 74.2% of the average relative abundance in each of the six species. The percentage of anti-*Bd* OTUs, represented as a fraction of the total number of OTUs recorded from each frog species, showed variation. The highest percentage of anti-*Bd* bacterial OTUs were present on *G. asterops* (171/216, 79.1% anti-*Bd* OTUs), followed by *Raochestes chlorosomma* (42/57, 73.7% anti-*Bd* OTUs), *D. melanostictus* (115/195, 58.9% anti-*Bd* OTUs), *B. beryet* (37/78, 47.4% anti-*Bd* OTUs), *L. doriae* (26/98, 26.5% anti-*Bd* OTUs), and *N. poocha* (20/101, 19.8% anti-*Bd* OTUs). Six anti-*Bd* bacterial OTUs, namely, *Stenotrophomonas* sp. (OTU 3), Sphingomonadales_unclassified (OTU 6), *Staphylococcus*sp. (OTU 7), Microbacteriaceae_unclassified (OTU 18), Micrococcaceae_unclassified (OTU 21), and Caulobacteraceae_unclassified (OTU 34), were present in all six frog species. They matched with *Silverstoneia flotator* (inhibitory_30), Sphingomonadaceae bacterium (KU738962.1), *Boophis madagascariensis* (ns_6), *Colostethus panamensis* (inhibitory_2), *Alytes obstetricans* (ns_64), and Caulobacteraceae bacterium (KU738923.1), respectively, from Woodhams et al. [15] and Muletz et al. [16] database. The average relative abundance of these OTUs was 17.8%. Percentage of anti-*Bd* bacterial OTUs, represented as a fraction of the total anti-*Bd* OTUs in different bacterial phyla were: Proteobacteria (175/285, 61.4% anti-*Bd* OTUs), Bacteroidetes (57/285, 20% anti-*Bd* OTUs), Actinobacteria (36/285, 12.3% anti-*Bd* OTUs), and Firmicutes (17/285, 5.96% anti-*Bd* OTUs).

Globally, frog skin microbiomes are replete with Proteobacteria, Bacteroidetes, and Actinobacteria [17–19]. Proteobacteria is also abundant in soils [20], and thereby, associated with tropical frog skins [17, 21], and majority of them (80% of the total OTUs present) are in the anti-*Bd* databases [15]*.* Bacterial family Pseudomonadaceae (Proteobacteria) has been reported from several frogs’ skin microbiomes with varying abundance [19]. However, this bacterial family was not found in *B .beryet*, *L. dorie*, *N. poocha*, and *R. chlorosomma* (Fig. 1b). Family Xanthomonadaceae (Proteobacteria) was dominant in the bacterial community in all the frog species, except *N. poocha*. Actinobacteria was dominant (49.12% relative abundance), and Proteobacteria was the least represented (6.98% relative abundance) in *N. poocha.* Actinobacteria are associated with freshwater ecosystems [22], and *N. poocha* being a mountain stream-dwelling frog shares the same habitat with the bacterial phylum. The differences in the bacterial phyla composition on *N. poocha* could also be due to seasonal differences, or changes in pH of water in streams [23].

In terrestrial frogs (*L. doriae* and *R. chlorosomma*), OTUs were higher than in aquatic (*N. poocha*) and arboreal (*G. asterops* and *B. beryet*) frogs (Table 1). A similar pattern has been documented in a global comparison of frog skin microbiomes [19]. Bacterial OTUs in terrestrial frogs could be attributed to their contact with soil, which has a rich bacterial community. Based on bacterial OTUs and their abundance, there was no consistent association between the niche of the frogs and their skin bacterial community composition (Fig. 1a). This lack of association suggests that frog skin bacterial community might not be an accurate descriptor of the niche of the frog.

Anti-*Bd* bacterial OTUs (51.7%) were higher than those reported on frog species from Costa Rica (13%) [17] and Panama (8.45%) [23]. Salamander skin microbiome from the Eastern USA has revealed high anti-*Bd* bacterial OTU abundance (87% average relative abundance), which was contributed by 13% of anti-*Bd* OTUs [18]. Other bacterial genera with anti-*Bd* OTUs on the frogs were: *Chryseobacterium* (23/285, 8% anti-*Bd* OTUs), *Elizabethkingia* (18/285, 6.3% anti-*Bd* OTUs), *Stenotrophomonas* (18/285, 6.3% anti-*Bd* OTUs), and *Pseudomonas* (13/285, 4.5% anti-*Bd* OTUs; see Supplemental file S2). These bacteria have been shown to exhibit anti-*Bd* activity in vitro [24–27]. Anti-*Bd* genera, namely, *Streptomyces* [26], *Propionibacterium*, *Microbacterium*, and *Micrococcaceae* [27], belonging to the phylum Actinobacteria, also possess anti-fungal properties. They were found in the skin microbiome of the six species of frogs examined. Since a large number of anti-*Bd* OTUs were detected, we hypothesize that a similar community of bacteria might be present in frog populations in the region.

Low abundance of anti-*Bd* bacteria on frog’s skin has been linked to high prevalence of *Bd* in the hotspots of infection [23, 28]. However, currently, there is no evidence suggesting that a high abundance of anti-*Bd* bacteria on frog’s skin is associated with low prevalence of *Bd* infection in coldspots of infection. A rich anti-*Bd* bacterial community that thrives on the skin of frogs has been revealed through this study. It could be one of the reasons for the low prevalence of *Bd* in frog populations in the region. Anti-*Bd* bacteria provide frogs with protection from *Bd* infections in different ways: (i) by producing anti-*Bd* metabolite [29], (ii) by producing biofilm on frog skin surface [1], and (iii) by host-mediated selection of anti-*Bd* metabolite producing bacteria on its skin [30]. Since frog skin microbiome reports are scarce from Asia, future studies should focus on role of skin bacterial community in affording protection from *Bd* infection by attenuation of the pathogen [31]. Bacterial flora in the soil or stream might be an important source for the frog skin microbiome; therefore, we hypothesize that *Bd* might be experiencing strong selection pressure both in the environment, and on the frogs in coldspots of infection.

## Supplementary Information

Supplementary file S1 **:** It contains a detailed description of methods used in generating the data and analyzing it. (DOCX 29 kb)

Supplementary file S2 **:** It provides the taxonomic identity of 551 OTUs obtained from the frog skin microbiome. (XLSX 65 kb)

## Data Availability

Sequencing files have been submitted at the NCBI Sequence Read Archive (SRA) repository (BioProject ID: PRJNA639992; Temporary Submission ID: SUB7618771).
