# Supplementary material for: Bacterial Community in the Skin Microbiome of Frogs in a Coldspot of Chytridiomycosis Infection
Source: Microb Ecol. 2021 Jan 13;82(2):554–8. doi: 10.1007/s00248-020-01669-5 (PMC8384794; doi:10.1007/s00248-020-01669-5)
Supplement: Supplementary file 1 — : It contains a detailed description of methods used in generating the data and analyzing it. (DOCX 29 kb) [file 248_2020_1669_MOESM1_ESM.docx]

Bacterial community in the skin microbiome of frogs in a coldspot of chytridiomycosis infection

Running title: frog skin microbiome

AUTHORS:

**Milind C. Mutnale, Gundlapally S. Reddy, Karthikeyan Vasudevan***

Authors Affiliations: CSIR-Centre for Cellular and Molecular Biology, Laboratory for the Conservation of Endangered Species, Hyderabad, Telangana, India.

***Corresponding Author:**

**Karthikeyan Vasudevan**, CSIR-Centre for Cellular and Molecular Biology, Laboratory for the

Conservation of Endangered Species, Hyderabad, Telangana, India.**karthik@ccmb.res.in**

**Supplementary methods:**

All anurans studied are hereafter referred to as frogs. A total of 18 samples comprising 6 frog species (Table 1) namely: *Blythophryne beryet* (*N*=3), *Duttaphrynus melanostictus* (*N*=3)*, Ghatixalus asterops* (*N*=3)*, Nyctibatrachus poocha* (*N*=3)*, Raorchestes chlorosomma* (*N*=3)*, and Limnonectes doriae* (*N*=3) were sampled between 2013 and 2015. Detailed methodology of sample collection, processing, and *Bd* diagnosis is provided in Mutnale et al [1]. In Brief, a total DNA was extracted from each sample and tested individually using nested PCR for *Bd* infection, and found to be negative [1]. Prevalence of *Bd* the populations of six frog species was calculated based on data from Mutnale et al., (2018). Frog skin swab DNA samples were initially subjected to full-length bacterial 16S rRNA gene PCR amplification with universal bacterial primer 27F (5'-AGAGTTTGATCCTGGCTCAG-3') and 1493R (5'-GGTTACCTTGTTACGACTT-3') with following conditions: initial denaturation at 95°C for 5 min, followed by 40 cycles of denaturation at 95 °C for 30 s, annealing at 55 °C for 30 s, extension at 72 °C for 30 s, and a final extension at 72 °C for 10 min. We used nested PCR targeting V3-V5 region using 338F (5-CCATCTCATCCCTGCGTGTCTCCGAC-TCAG-ACGAGTGCGT-CACCTACGGGTGGCAGC-3) and 909R (CCTATCCCCTGTGTGCCTTGGCAGTC-TCAG-CCGTCAATTYHTTTRAGT) primer set [2]. The short amplicons were removed using Agencourt AM Pure PCR purification kit (Beckman Coulter, USA), DNA quantity and quality was assessed on Quant-iTPico Green dsDNA Assay Kit (Invitrogen) and Agilent Bioanalyzer 2100 DNA 7000 chip (Agilent, USA) respectively. The amplicon library was then subjected to emulsion PCR using the GS-FLX Titanium Sequencing kit (Roche, Nutley, NJ, USA) and then sequenced using Roche GS-FLX system. Amplified PCR products were pooled according to frog species and unique barcodes were used for each pooled sample (*B. beryet-* ATATCGCGAG, *D. melanostictus*-AGCACTGTAG, *G. asterops-* ACGAGTGCGT, *L. doriae-* ATCAGACACG, *N. poocha-* ACGCTCGACA, and *R. chlorosomma-* AGACGCACTC). For analysis of sequenced data, software mothur v.1.41.3 [3], was used to perform denoising, removing of low quality, chimeric reads sequences, and alignment of our frog microbiome Operational Taxonomic Unit (OTU) data. We used the SILVA version 123 database [4, 5] for taxonomic assignment of OTUs. We used “dist.seqs” command in mothur to make distance matrix with 0.15 cut-off, and used “cluster” command at the cut-off 0.03 (≥ 97%) to assign sequences to OTUs. Software microbiomeanalyst [6] was used to analyze the output generated from mothur. We rarified all samples to the smallest number of sequences obtained from a frog species to compare OTU diversity among the host species using package vegan [7] in R v 3.6.3 [8]. Using the same package in R, we also performed a cluster analysis using Bray-Curtis measure to find the relationship between the frog skin microbiome, and their host guild, using “vegdist” and “hclust” function. In order to estimate the abundance of the anti-*Bd* bacterial community on frog skin, all the OTUs were searched using custom BLAST option in Geneious software 8.1 (Biomatters, available from https://www.geneious.com/) in the anti-*Bd* bacterial database [9, 10] with the cut-off set at ≥ 97% sequence similarity.

**Reference:**

1. Mutnale MC, Anand S, Eluvathingal LM, et al (2018) Enzootic frog pathogen *Batrachochytrium dendrobatidis* in Asian tropics reveals high ITS haplotype diversity and low prevalence. Sci Rep 8:10125. https://doi.org/10.1038/s41598-018-28304-1

2. Ram JL, Karim AS, Sendler ED, Kato I (2011) Strategy for microbiome analysis using 16S rRNA gene sequence analysis on the Illumina sequencing platform. Syst Biol Reprod Med 57:162–170. https://doi.org/10.3109/19396368.2011.555598

3. Schloss PD, Gevers D, Westcott SL (2011) Reducing the Effects of PCR Amplification and Sequencing Artifacts on 16S rRNA-Based Studies. PLoS One 6:e27310. https://doi.org/10.1371/journal.pone.0027310

4. Quast C, Pruesse E, Yilmaz P, et al (2012) The SILVA ribosomal RNA gene database project: improved data processing and web-based tools. Nucleic Acids Res 41:D590–D596. https://doi.org/10.1093/nar/gks1219

5. Yilmaz P, Parfrey LW, Yarza P, et al (2014) The SILVA and “All-species Living Tree Project (LTP)” taxonomic frameworks. Nucleic Acids Res 42:D643–D648. https://doi.org/10.1093/nar/gkt1209

6. Dhariwal A, Chong J, Habib S, et al (2017) MicrobiomeAnalyst: a web-based tool for comprehensive statistical, visual and meta-analysis of microbiome data. Nucleic Acids Res 45:W180–W188. https://doi.org/10.1093/nar/gkx295

7. Oksanen J, Blanchet FG, Friendly M, et al (2019) vegan: Community Ecology Package. R package Version 2.5-6 2019

8. TEAM RDC (2013) R: A language and environment for statistical computing

9. Woodhams DC, Alford RA, Antwis RE, et al (2015) Antifungal isolates database of amphibian skin-associated bacteria and function against emerging fungal pathogens. Ecology 96:595–595. https://doi.org/10.1890/14-1837.1

10. Muletz-Wolz CR, DiRenzo G V., Yarwood SA, et al (2017) Antifungal bacteria on woodland salamander skin exhibit high taxonomic diversity and geographic variability. Appl Environ Microbiol 83:e00186-17. https://doi.org/10.1128/AEM.00186-17
